# Enhancement of Resistive and Synaptic Characteristics in Tantalum Oxide-Based RRAM by Nitrogen Doping

**DOI:** 10.3390/nano12193334

**Published:** 2022-09-24

**Authors:** Doohyung Kim, Jihyung Kim, Sungjun Kim

**Affiliations:** Division of Electronics and Electrical Engineering, Dongguk University, Seoul 04620, Korea

**Keywords:** RRAM, TaO_x_, nitrogen doping, resistive switching, variability, potentiation, depression

## Abstract

Resistive random–access memory (RRAM) for neuromorphic systems has received significant attention because of its advantages, such as low power consumption, high–density structure, and high–speed switching. However, variability occurs because of the stochastic nature of conductive filaments (CFs), producing inaccurate results in neuromorphic systems. In this article, we fabricated nitrogen–doped tantalum oxide (TaO_x_:N)–based resistive switching (RS) memory. The TaO_x_:N–based device significantly enhanced the RS characteristics compared with a TaO_x_–based device in terms of resistance variability. It achieved lower device–to–device variability in both low-resistance state (LRS) and high–resistance state (HRS), 8.7% and 48.3% rather than undoped device of 35% and 60.7%. Furthermore, the N–doped device showed a centralized set distribution with a 9.4% variability, while the undoped device exhibited a wider distribution with a 17.2% variability. Concerning pulse endurance, nitrogen doping prevented durability from being degraded. Finally, for synaptic properties, the potentiation and depression of the TaO_x_:N–based device exhibited a more stable cycle–to–cycle variability of 4.9%, compared with only 13.7% for the TaO_x_–based device. The proposed nitrogen–doped device is more suitable for neuromorphic systems because, unlike the undoped device, uniformity of conductance can be obtained.

## 1. Introduction

New technologies such as artificial intelligence (AI) and the Internet of Things (IoT) are gaining attention, so rapid processing of vast amounts of data and information is required. However, in traditional digital computing—the von Neumann architecture—operation and storage devices are separated. Accordingly, bottlenecks occur when transferring complex data between devices [1,2]. Therefore, neuromorphic computing has emerged because of its parallel data processing with low power consumption and high-density structure. A neuromorphic system mimics a biological system—the human brain [3,4,5]. Recently, oxide–based device and resistance–based random–access memory designs, such as magnetic random–access memory (MRAM), ferroelectric random–access memory (FRAM), phase–change random–access memory (PRAM), spin–torque–transfer random–access memory (STT–RAM) and resistive random–access memory (RRAM) have been studied for implementation in neuromorphic systems [6,7,8]. RRAM is a promising candidate for the next generation of neuromorphic systems because of advantages such as high endurance, high–speed switching, low–power operation, scaling down capability, and multi–level cell (MLC) capability [9,10,11,12].

Despite these advantages, the filament–type RRAM cannot avoid large variations in conductance because the formation and rupture of filaments occur randomly, a drawback for use in neuromorphic systems [13]. Several studies have been reported to suppress the probabilistic nature of conducting filament formation, such as combining several oxide layers [14], semiconducting oxides [15,16], and doping techniques [17]. Nitrogen doping has been widely studied because it can control conductance accurately and effectively. Ref. [18] reported that N–doping in Ti/TiO_x_/Pt enhances the reliability effect on neuromorphic systems. Refs. [19,20,21,22,23] demonstrated that nitrogen doping in insulators can reduces the leakage path. Arikado, T et al., [24] stated that nitrogen eliminates oxygen vacancy related gap states by changing the charged states of V_o_ to V_o_^2+,^ so the leakage path can be reduced. Another important effect of nitrogen in the oxide layer is the restriction of oxygen ions’ diffusion. Y. E. Syu et al., [25] stated that, due to the higher bonding energy of N–O bond than the O–O bond, nitrogen can capture the oxygen atom to localize the oxygen ion near the conducting filament. Misha et al. [26] reported that incorporating nitrogen in TaO_x_ improves uniformity at a low operating current. The performance of N–doped memory devices compared with undoped memory device in previous reports is summarized in Table 1.

In this study, the effect of nitrogen doping on synaptic properties and DC properties was also examined, in contrast with previous studies [26]. By incorporating nitrogen, smaller variations in DC endurance, AC endurance, device–to–device resistance, and set voltages are achieved compared with the undoped film. Furthermore, conductance is efficiently and gradually modulated by pulse–train measurement in the N–doped TaO_x_. Therefore, we investigated enhancing RS and synaptic properties in TaO_x_ films with nitrogen doping.

## 2. Materials and Methods

The Ta/TaO_x_/Pt and Ta/TaO_x_:N/Pt devices were fabricated using the following process. First, we deposited the platinum (Pt) bottom electrode (BE) with a thickness of 100 nm using a thermal evaporator on a silicon oxide–silicon (SiO_2_/Si) substrate. The substrate was cleaned with acetone, isopropyl alcohol (IPA), and deionized (DI) water under ultra-sonication for 5 min each. The tantalum oxide (TaO_x_) and nitrogen–doped tantalum oxide (TaO_x_:N) switching layers were deposited with a thickness of 50 nm at room temperature. We applied a radiofrequency (RF) sputtering power of 240 W with the 3–inch tantalum metal target.

The pressure in the main chamber was maintained at 5 mTorr, and the gas flow rate was set to 20 sccm of Ar gas, 6 sccm of O_2_ gas, and an additional 1 sccm of N_2_ gas for the TaO_x_:N device. We then coated the negative PR and patterned it with a square pattern size of 100 μm. Then, the Ta top electrode (TE) was deposited by DC sputtering with a thickness of 100 nm.

**Figure 1:**
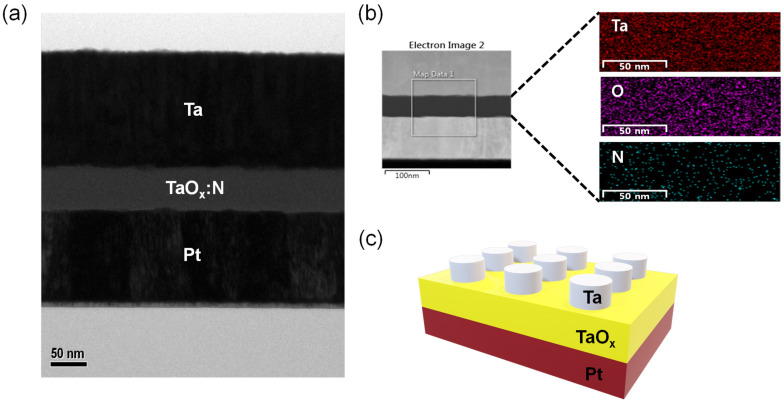
(**a**) High magnification imaged by TEM. (**b**) Overall image for EDS mapping. (**c**) Schematic displaying the device structure.

Figure 1a demonstrates the cross–sectional transmission electron microscope (TEM, KANC, Suwon 16229, Republic of Korea) view and Figure 1b displays the distribution of the nitrogen doped insulator components (Ta, O, N) as indicated by EDS mapping. Figure 1b shows that the N content is uniformly distributed, indicating uniform and shallow N–doping on the TaO_x_ films. Figure 1c illustrates the final patterned structure after the TE lift–off process. We examined the microstructure and thickness analysis of the memristor device. A semiconductor parameter analyzer (Keithly 4200–SCS and PMU ultrafast mode, Tektronix Inc., Beaverton, OR 97077, USA) was used to evaluate the device’s electrical characteristics.

## 3. Results and Discussion

**Figure 2:**
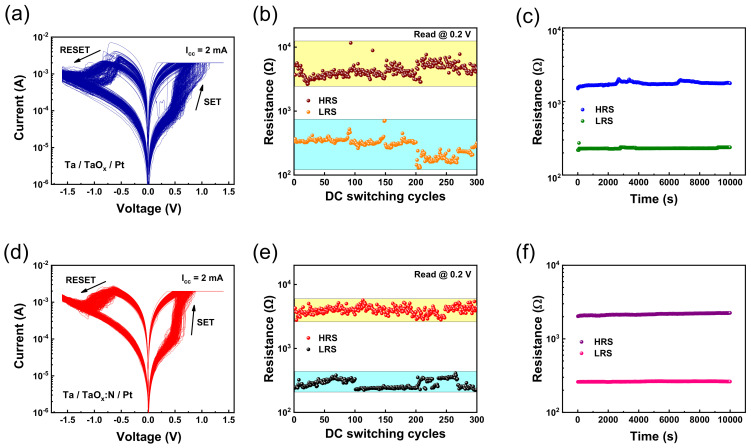
RS characteristics of Ta/TaO_x_/Pt device: (**a**) I−V curves of 300 consecutive switching cycles, (**b**) LRS and HRS state resistance distribution for each cycle at 0.2 V (**c**) Retention characteristics test for LRS and HRS. RS characteristics of Ta/TaO_x_:N/Pt device: (**d**) I−V curves of 300 repetitive switching cycles, (**e**) LRS and HRS state resistance measurement for each cycle at 0.2 V (**f**) Retention characteristics test for LRS and HRS.

First, the electrical properties of the Ta/TaO_x_/Pt and Ta/TaO_x_:N/Pt memristors were measured. A preliminary forming process was required to produce a low–resistance state (LRS) from the initial state [27,28]. Both devices conducted 300 consecutive ON/OFF cycles by positive set and negative reset for the bipolar RS (BRS) after the forming process, as depicted in Figure 2a,d. Each memristor was measured under a compliance current of 2 mA in the set process with no current limit during reset switching to ensure a fair comparison. Furthermore, a negative voltage of −1.6 V was applied to switch from the LRS to the high–resistance state (HRS), and HRS changed to LRS under a positive voltage sweep from 0 to 1.4 V.

The detailed distribution of HRS and LRS values were extracted from the I−V characteristics measured at 0.2 V (V_read_) during a cyclic test, as depicted in Figure 2b,e. The TaO_x_–based memristor exhibited a significant difference in resistance in the set and reset operations with cycle–to–cycle resistance variabilities (σ/μ) of 27.8% (LRS) and 23.7% (HRS). However, the TaO_x_:N–based memristor exhibited a stable RS characteristic with relatively small cycle–to–cycle resistance variabilities of 15.7% (LRS) and 13.2% (HRS). A retention characteristic test was performed to confirm the performance of the TaO_x_–based and TaO_x_:N–based memristors, as depicted in Figure 2c,f. Both devices can be maintained for 10^5^ s without any degradation.

**Figure 3:**
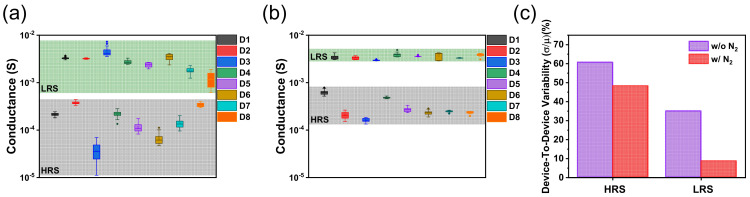
HRS and LRS distributions of eight randomly selected memory cells of (**a**) Ta/TaO_x_/Pt device and (**b**) Ta/TaO_x_:N/Pt device. (**c**) Device–to–device variation for two devices.

Eight randomly selected cells were assessed to confirm the device–to–device conductance uniformity. Uniformity is also to do with the size of the possible formed clusters, and the size of both devices is equal [29]. Each cell of both devices was applied by a voltage sweep in the range of 0 to −1.6 and 0 to 1.4 V for 20 cycles with a voltage step of 0.01 V, as presented in Figure 3a, b. For the TaO_x_–based device, the HRS ranges from 11.2 to 437 μS, and the LRS ranges from 0.62 to 7.3 mS (Figure 3a). In contrast, HRS values from 134 to 784 μS and LRS values from 3 to 4.9 mS are observed in the TaO_x_:N–based device (Figure 3b).

For the TaO_x_–based device, the range of conductance levels was wider than for the TaO_x_:N–based device. We used resistance variability to numerically identify the distribution of the HRS and LRS and accurately examine the extent to which data points differ. When the variability was calculated by considering only the average of LRS and HRS values in each cell, the device–to–device HRS variability decreased from 60.7 to 48.3%, and that of LRS decreased from 35 to 8.7% when nitrogen doping was applied, as illustrated in Figure 3c.

**Figure 4:**
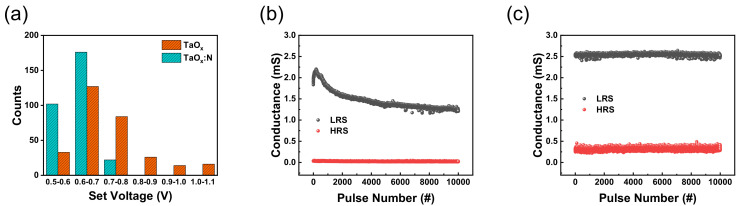
(**a**) Statistical distribution of set voltages for Ta/TaO_x_/Pt and Ta/TaO_x_:N/Pt. Pulse endurance characteristics of (**b**) Ta/TaO_x_/Pt device and (**c**) Ta/TaO_x_:N/Pt device.

Furthermore, the set voltage distribution of both devices was characterized in histograms, as plotted in Figure 4a. 

The uniformity of the set voltages is crucial to ensure error–free operation. Set voltage is the threshold where the resistance of the I−V curve abruptly decreases from HRS to LRS. As illustrated in Figure 4a, a wider set voltage distribution of the TaO_x_–based device is observed in the range of 0.5 to 1.1 V. In contrast, the TaO_x_:N–based device exhibits a more concentrated distribution in the range of 0.5 to 0.8 V. The statistical distribution of set voltages distribution is summarized in Table 2.

From the results of the DC characteristics, as described previously in Figure 2, Figure 3 and Figure 4, we confirmed that nitrogen doping on the TaO_x_–based device improved resistance variability and reliability—the most critical for ReRAM device applications [30].

Furthermore, a pulse endurance test was performed for up to 10^5^ cycles to compare AC characteristics. A 10-μs pulse width of both set and reset pulses and amplitudes of 1.45 and −1.7 V were applied to both devices for a reliable comparison. The TaO_x_–based memristor exhibited unstable RS operation. Moreover, the conductance value in LRS degraded throughout the measurement, as presented in Figure 4b. In contrast, the TaO_x_:N–based memristor exhibited excellent endurance of up to 10^5^ cycles.

**Figure 5:**
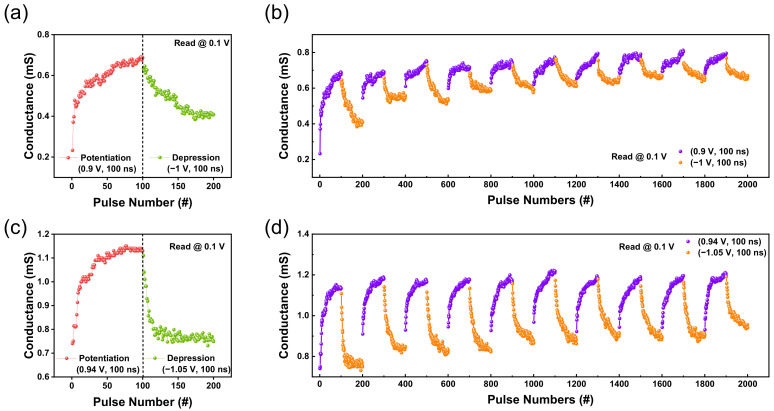
Ta/TaO_x_/Pt device: (**a**) LTP and LTD are established with 100 identical write pulses (amplitudes of 0.9 V, the pulse width of 100 ns) and erase pulses (amplitudes of −1 V, the pulse width of 100 ns) (**b**) Ten cycles of potentiation and depression (potentiation first). Ta/TaO_x_:N/Pt device: (**c**) LTP and LTD are established with 100 identical write pulses (amplitudes of 0.94 V, the pulse width of 100 ns) and erase pulses (amplitudes of −1.05 V, the pulse width of 100 ns) (**d**) Ten cycles of potentiation and depression (potentiation first).

Synaptic plasticity, such as long–term potentiation (LTP) and long–term depression (LTD), are crucial aspects of the application of neuromorphic systems [31]. The LTP and LTD were measured and discussed to compare the synaptic properties between the two devices. In Figure 5a,c, 100 potentiation and depression pulses were applied with 0.1 V read voltage pulses. The amplitude of potentiation and depression pulses were set to 0.9 and −1 V for the TaO_x_–based memristor and 0.94 and −1.05 V for the TaO_x_:N–based memristor. Moreover, the pulse width was fixed to 100 nS to ensure the pulse conditions were as similar as possible while modulating conductance gradually. 

Ten cycles of potentiation and depression were conducted with these consecutive pulses. Figure 5b illustrates the degradation of the potentiation and depression during cycles of the undoped device. In contrast, Figure 5d illustrates a more constant and stable pulse measurement without degradation. When calculating the cycle–to–cycle resistance variability between the two devices, the nitrogen–doped device exhibited less variability of 4.9% compared with the 13.7% of the TaO_x_–based memristor. 

**Figure 6:**
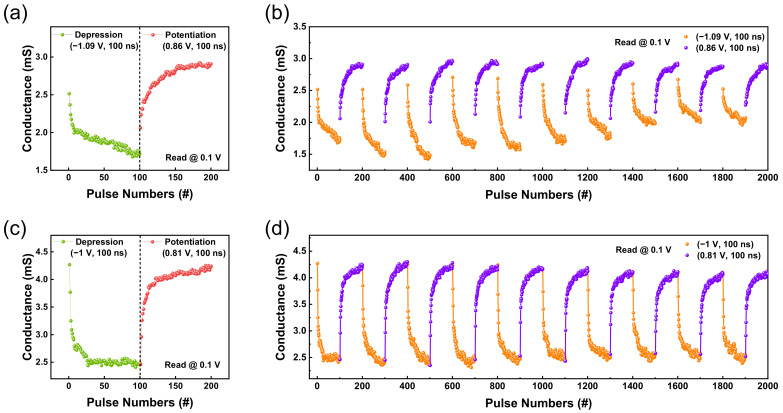
Ta/TaO_x_/Pt device: (**a**) LTD and LTP are established with 100 identical erase pulses (amplitudes of −1.09 V, the pulse width of 100 ns) and write pulses (amplitudes of 0.86 V, the pulse width of 100 ns) (**b**) Ten cycles of depression and potentiation (depression first). Ta/TaO_x_:N/Pt device: (**c**) LTD and LTP are established with 100 identical erase pulses (amplitudes of −1 V, the pulse width of 100 ns) and write pulses (amplitudes of 0.81 V, the pulse width of 100 ns) (**d**) Ten cycles of depression and potentiation (depression first).

Even for nitrogen–doped devices (4.9%), cycle–to–cycle variability occurred, as depicted in Figure 5. We reduced this variability by applying a DC set voltage to produce LRS. We then proceeded first with depression rather than potentiation, as depicted in Figure 6a,c. As depicted in Figure 6, 100 depression and potentiation pulses were applied with a 0.1 V read voltage pulse; ten cycles were conducted. The amplitudes of depression and potentiation pulses were set to −1.09 and 0.86 V for the TaO_x_–based memristor and −1 and 0.81 V for the TaO_x_:N–based memristor; the pulse width was set to 100 nS. 

As depicted in Figure 6b, the cycle–to–cycle variation issue still occurred in the TaO_x_–based device. The conductance of the last point of a cycle does not match the first point of a subsequent cycle. In contrast, Figure 6d illustrates more stable synaptic properties, with a 2.1% cycle–to–cycle variability—a smaller value than when potentiation was performed first (4.9%). Based on these pulse measurement results (Figure 5 and Figure 6), device reliability is increased by incorporating nitrogen—similar to the DC measurement results. In biological neural networks, calculation and storage of information are performed simultaneously [32], so uniformity of resistance value is critical. Consequently, the TaO_x_:N–based memristor, which has higher cycle–to–cycle uniformity, is suitable for neuromorphic computing applications. Key performance indicators achieved in the present study are summarized in Table 3.

## 4. Conclusions

In this study, we investigated the role of nitrogen doping on resistive and synaptic characteristics while comparing the Ta/TaO_x_/Pt and Ta/TaO_x_:N/Pt devices. The TaO_x_:N–based memristor exhibited uniformity of resistance state and set voltages and a 10^5^ pulse endurance. Furthermore, synaptic properties such as potentiation and depression were conducted with a pulse train. The TaO_x_:N–based memristor exhibited more stable conductance modulation when measuring potentiation first and depression first. In contrast, the TaO_x_–based memristor produces variability in the I–V curves, set voltages, and device-to-device resistance. It also degrades when pulse endurance, potentiation, and depression are measured. When we use RRAM as a neuromorphic system, the device should have low resistance variability. Therefore, a TaO_x_:N–based device is more suitable for an artificial synapse than an undoped–TaO_x_ device.

## Figures and Tables

**Table 1:** Performance of N–doped Memory Devices Compared with Undoped Memory Device in Previous Reports.

Device	Doping	On/Off Ratio	V_set_ (V)	Retention (s)	MNIST	Ref
Pt/NbO_x_/Pt	Undoped	~10	1.3–1.9	4 × 10^3^	N.A.	[20]
	N–doped	~10^3^	0.4–1.3	6 × 10^4^	N.A.	
Ti/WO_x_/Pt	Undoped	~10	N.A.	10^2^	N.A.	[19]
	N–doped	~10^2^	N.A.	10^4^	N.A.	
Ti/TiO_x_/Pt	Undoped	-	Not uniform	10^3^	21.1%	[18]
	N–doped	-	uniform	10^5^	64.4%	

**Table 2:** Statistical Distribution of Set Voltages Obtained With 300 DC Switching Cycles.

	V_set_ (V)		
Device	Μ	σ	σ∕μ
Undoped	0.72	0.12	17.2%
N–doped	0.62	0.059	9.4%

**Table 3:** Key Performance Indicators Achieved in The Present Study.

Device	Cycle-to-Cycle Variability (LRS, HRS)	Retention	Device-to-Device Variability (LRS, HRS)	V_set_ Variability	Pulse Endurance	LTP, LTD Variability
Undoped	(27.8%, 23.7%)	~10^4^ s	(35%, 60.7%)	17.2%-	degradation	4.9%
N–doped	(15.7%, 13.2%)	~10^4^ s	(8.7%, 48.3%)	9.4%	~10^5^	13.7%

## Data Availability

Not applicable.
